# Nutrition-Related Mobile Application for Daily Dietary Self-Monitoring

**DOI:** 10.1155/2022/2476367

**Published:** 2022-08-30

**Authors:** Maria Ulfa, Winny Setyonugroho, Tri Lestari, Esti Widiasih, Anh Nguyen Quoc

**Affiliations:** ^1^Master of Hospital Administration, Postgraduate Program, Universitas Muhammadiyah Yogyakarta, Kasihan, Indonesia; ^2^Information Technology Department, Politeknik Negeri Padang, Padang, Indonesia; ^3^Medical Education Program, Faculty of Medicine, Universitas Muhammadiyah Semarang, Semarang, Indonesia; ^4^Department of Food Microbiology and Molecular Biology, National Institute of Nutrition, Hanoi, Vietnam

## Abstract

Nutrition apps for mobile devices such as smartphones are becoming more widely available. They can help ease the arduous chore of documenting intake for nutritional assessment and self-monitoring. This allows people to control food intake, support their participation in physical activities, and promote a healthy lifestyle. However, there remains a lack of research regarding systematic analysis mapping studies in this area. The objective of this study is to identify dietary self-monitoring implementation strategies on a mobile application. This study analyzed 205 journals from the Scopus database using the descriptive-analytic method. The records used in this exploration study were those released between 2007 and 2021 that were collected based on the keywords “dietary self-monitoring,” or “nutrition application,” or “nutrition apps,” and “calorie application.” Data analysis was conducted using the VOSviewer and NVivo software analytical tools. The results show that research studies on dietary self-monitoring increased in 2017. Results also indicated that the country that contributed the most to this topic was China. The study on mobile applications for dietary self-monitoring revealed seven clusters of dominant themes: attitude to improved dietary behaviors, parameters for disease diagnosis, noncommunicable diseases, methods, nutrition algorithms, mobile health applications, and body mass index. This study also analyzed research trends by year. The current research trends are about dietary self-monitoring using a mobile application that can upgrade people's lifestyles, enable real-time meal recording and the convenience of automatically calculating the calorie content of foods consumed, and potentially improve the delivery of health behavior modification interventions to large groups of people. The researchers summarized the recent advances in dietary self-monitoring research to shed light on their research frontier, trends, and hot topics through bibliometric analysis and network visualization. These findings may provide valuable guidance for future research and perspectives in this rapidly developing field.

## 1. Introduction

Nowadays, many people are aware of their health and the importance of healthy lifestyle. Nutritional knowledge is essential for promoting good eating habits since it ensures that necessary nutrient requirements are met to avoid malnutrition [1]. By exposing people to education-based interventions such as improving nutrition knowledge, one is likely to improve dietary behaviors and food choices as well [2–4]. People who understand the link between specific health issues and poor nutrition are better equipped to track and manage their weight through their diet choices. There is a connection between an adult's understanding of dietary requirements and good eating habits [5]. Thus, awareness of the need for healthy eating habits and understanding the foods to eat are the first steps toward changing one's eating habits.

Food packets (both on the front and back of the product), food commercials, and restaurant menus all provide nutritional information of the food or meal they offer [6, 7]. One of the fundamental purposes of providing nutritional information in the food selection environment regarding public health is to raise awareness and understanding about the nutritional composition of food products. This information could lead to more significant purchases and healthier foods, ultimately contributing to improved health [8]. According to previous studies, most consumers rely on easily accessible sources of information for nutrition information, such as nutrition labels [9, 10].

Self-monitoring is a common way to diet and achieves weight loss, particularly the self-monitoring of physical activity (PA) and nutritional intake, which are essential components of behavioral weight control programs [11]. Existing research on self-monitoring found a substantial link between self-monitoring and weight loss in some or all three components evaluated in behavioral weight loss studies: diet, workout, and self-weighing [12–16]. Some researchers found that individual dietary self-monitoring has been regarded as one of the most effective techniques for maintaining body weight [11, 17, 18]. The reliance on reliable recollection, the lack of consistency in reporting, and the general cost of data logging hinder diet monitoring [19]. Moreover, self-monitoring diets necessitate making a daily log of all foods, their energy content, and other macronutrients the food contains such as fat grams [20]. This process can make a person's diet better controlled and consistent.

Nutrition and healthy lifestyle applications could be a low-cost and practical approach to spreading diet and nutrition information to the public. These apps also provide focused information to specific populations, such as overweight persons, cancer survivors, and people at risk for heart diseases [21]. With the advancement of mobile phone technology, various health-related mobile apps have been created and are now widely utilized to address health issues [22]. Health apps offer the potential to reduce some of the constraints that traditional behavior change prescriptions posed, such as cost, patient burden, and unpredictable adherence. Many apps give users additional tools for tracking their health or attaining health-related objectives [23]. The knowledge based on mobile devices for nutritional data collection and behavior change interventions has developed with much of it focusing on personal digital assistants (PDAs) [19].

Therefore, this study offers a bibliometric analysis of the advancements in research on dietary self-monitoring. It seeks to assess the intensity and dominance of research topics in the scientific community regarding this emerging phenomenon, focusing explicitly on dietary self-monitoring.

This article aims to provide primary data and to identify dietary self-monitoring implementation strategies on a mobile application for the use of advanced future research suggestions and provide recommendations for developing dietary self-monitoring applications.

## 2. Method

This research approach applied a qualitative literature review study. Research data were collected by searching the Scopus database (https://www.scopus.com/), an internationally recognized database of peer-reviewed journals. The searches in the Scopus database were conducted using the keywords “dietary self-monitoring” or “nutrition application,” or “nutrition apps,” or “calorie application,” with publication dates from 2007 until 2021.

**Figure 1:**
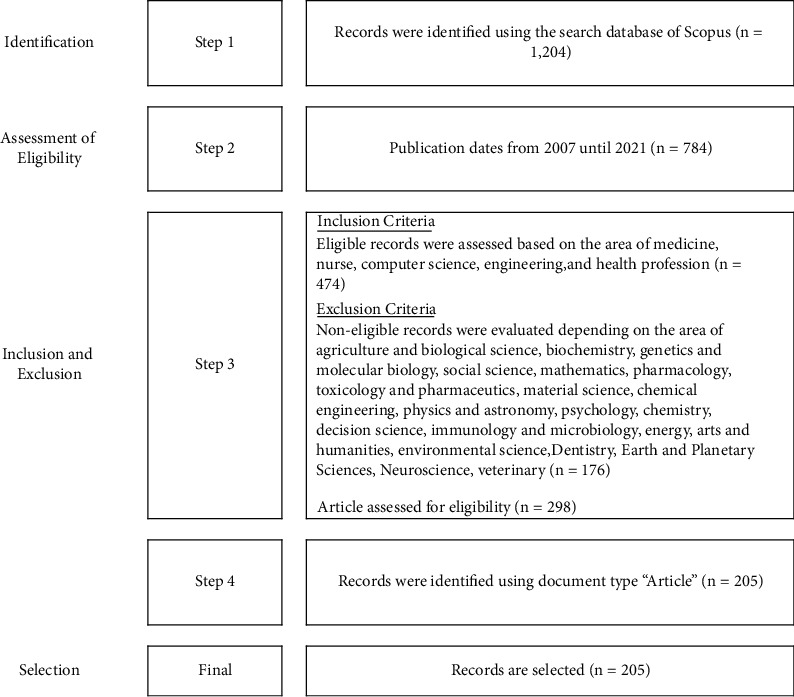
The steps of searching and selecting articles.

All data were taken during the same period, May 2022, to eliminate the bias caused by the increase in the database. The steps taken in this investigation are depicted in Figure 1 to give a clear picture of the study conducted. The step of collecting data was based on Nobanee et al. study [24]. Data was exported to RIS file format to distribute research map information. Then, the bibliometric leadership map was determined using three types of analysis: Scopus menu search results analysis, VOSviewer software analysis, and NVivo 12 Plus software analysis.

Scopus search results were analyzed using the descriptive method based on the year of publication, publishing institution, country of publication, name of publication, and research topic. Meanwhile, the use of VOSviewer determined a bibliometric map of research development based on the big subject of nutrition and adult. The collected data was refined several times to obtain the best information related to dietary self-monitoring. The NVivo software was used to test the correlation between indicators, variables, and keywords used in previous studies.

One finding was then drawn to serve as further research from this correlation. Furthermore, VOSviewer software was employed to map the most dominant keywords when studying dietary self-monitoring, to make further research into dietary self-monitoring applications. The context of dietary self-monitoring was based on the title or author keyword. Ultimately, the following query was conducted: (TITLE (dietary AND self AND monitoring) OR TITLE (nutrition AND application) OR TITLE (nutrition AND apps) OR TITLE (calorie AND application)) AND PUBYEAR >2006 AND PUBYEAR <2022 AND (LIMIT-TO (SUBJAREA, “MEDI”) OR LIMIT-TO (SUBJAREA, “NURS”) OR LIMIT-TO (SUBJAREA, “ENGI”) OR LIMIT-TO (SUBJAREA, “COMP”) OR LIMIT-TO (SUBJAREA, “HEAL”)) AND (EXCLUDE (SUBJAREA, “AGRI”) OR EXCLUDE (SUBJAREA, “BIOC”) OR EXCLUDE (SUBJAREA, “SOCI”) OR EXCLUDE (SUBJAREA, “MATH”) OR EXCLUDE (SUBJAREA, “PHAR”) OR EXCLUDE (SUBJAREA, “MATE”) OR EXCLUDE (SUBJAREA, “CENG”) OR EXCLUDE (SUBJAREA, “PHYS”) OR EXCLUDE (SUBJAREA, “PSYC”) OR EXCLUDE (SUBJAREA, “CHEM”) OR EXCLUDE (SUBJAREA, “DECI”) OR EXCLUDE (SUBJAREA, “ENER”) OR EXCLUDE (SUBJAREA, “IMMU”) OR EXCLUDE (SUBJAREA, “ARTS”) OR EXCLUDE (SUBJAREA, “ENVI”) OR EXCLUDE (SUBJAREA, “DENT”) OR EXCLUDE (SUBJAREA, “EART”) OR EXCLUDE (SUBJAREA, “NEUR”) OR EXCLUDE (SUBJAREA, “VETE”)) AND (LIMIT-TO (DOCTYPE, “ar”)). This query produced 205 documents.

Using bibliometric searches, the researchers sorted dimensions of analysis and units and for the bibliometric study, and mixed citations were deployed. The citations included co-authorships, which helped in examining the research field's social structure; a bibliographic coupling that utilized several references shared by two documents as comparison measure; co-occurrences to comprehend the document set patterns that underlie the research; and co-citations, which could help to determine the conceptual structure of the topic of the study. To generate and produce the figures and data from cited articles, the authors conducted co-occurrence analysis of keywords, co-authorship analysis of the influential authors and country distribution, co-citation analysis of cited sources, citation analysis of documents and organizations, and co-citation reference network analysis of dietary self-monitoring to generate the clusters/streams.

## 3. Result and Discussion

### 3.1. Publication by Year

**Figure 2:**
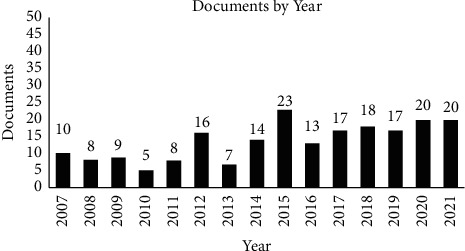
Publication by year.

In Figure 2, complete bibliometric research was led using the VOSviewer software and Scopus. The analysis of this study contained 205 documents from the year 2007 to 2021.

Figure 2 also illustrates the annual trend in publications related to dietary self-monitoring. Data for this study were collected from 2007 to 2021. In the previous 15 years, research studies on dietary self-monitoring have shown an increase in trend of the number of articles within the past ten years, especially in 2015.

Besides the number of publications each year, this study also analyzes the trends of research topics in this area. The most significant trends occurred from 2013 until 2017. In 2013, the trends were about nutritional support, algorithms, health knowledge, attitudes, and behavior. Nutritional policy, food preference, and health promotion were examined in 2014. Moreover, in 2015, nutritional assessment, risk factors, and self-care were trends. Obesity, nutrition therapy, and body weight were explained in 2016. Trends in 2017 until now have been mobile application, mHealth, and self-monitoring. This shows that study is improving each year.

Current trends are about dietary self-monitoring based on mobile applications wherein these mobile applications can upgrade people's lifestyles. In the study conducted by Turner-McGrievy et al., they were uncertain whether using a mobile app increased adherence to physical activity and dietary intake recommendations [25]. In addition, dietary self-monitoring via mobile devices may enable real-time meal recording and the convenience of automatically calculating the calorie content of foods consumed [25]. Smartphone applications can potentially improve the delivery of health behavior modification interventions to large groups of people, resulting in a favorable cost-utility ratio [26]. Furthermore, these devices will help those who wish to be healthy and change their lifestyles as measurement and feedback systems are becoming more refined and individualized [27].

### 3.2. Geographical Distribution

**Figure 3:**
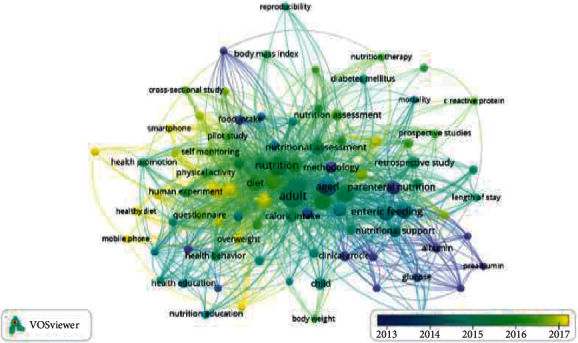
Overlay publication by year.

Several articles led to studies on healthcare professionals' nutritional self-monitoring in forty-one nations. China, the United States, the United Kingdom, Australia, Spain, Brazil, Canada, Germany, France, and Netherlands were geographically distributed to display scientific productivity by country (Figure 3) using “Microsoft Excel 365” to create the map. The different hues of blue signify different levels of productivity. Thus, the darker the shade of blue, the higher the output. China (*n* = 56), the United States (*n* = 52), and the United Kingdom (*n* = 19) were the top three countries producing the most documents.

**Figure 4:**
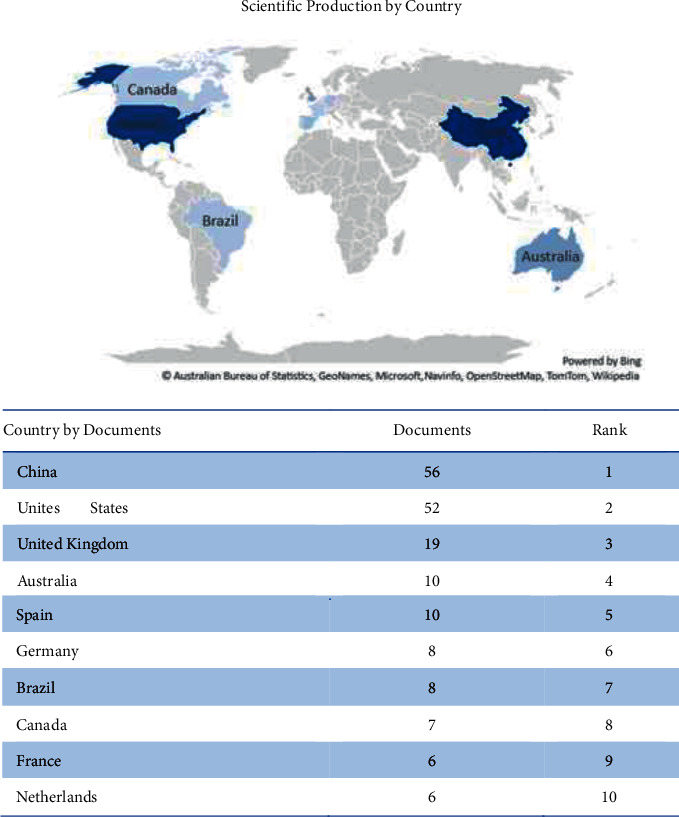
Documents by country.

**Figure 5:**
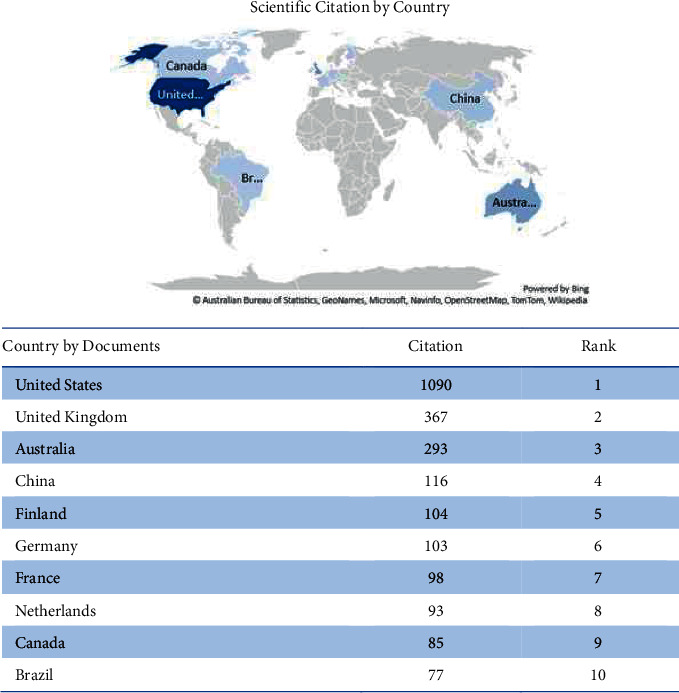
Documents by citations.

The top 10 countries are sorted based on the number of publications (Figure 4) and citations (Figure 5). Based on the ranking, China is placed first in dietary self-monitoring research and initiation yet placed fourth in citation by the government sector. Meanwhile, the United States is the country ranked second by documents and ranked first in the category of sources.

### 3.3. Subject Area

**Figure 6:**
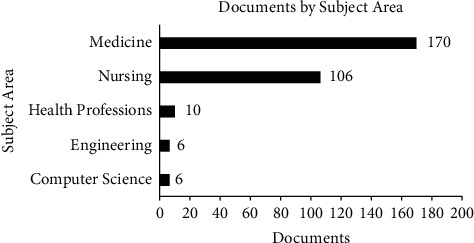
Documents by subject area.

This study also listed published journals based on their subject areas. Most dietary self-monitoring studies were in medicine, computer science, and engineering. The additional subject areas covered in this study are presented in Figure 6.

The reason why the researchers chose these subjects is to be more accurate when selecting the articles. These subject areas, including medicine, nursing, health profession, engineering, and computer science, are the subjects that are related to this research topic. Moreover, the articles in this selected subject areas are connected with one another. The medicine, nursing, and health profession subject areas represent the dietary and nutrition issue that will be discussed in this study. Furthermore, the engineering and computer science subject areas characterize the application, which is in line with this research study.

### 3.4. Keyword Analysis

**Figure 7:**
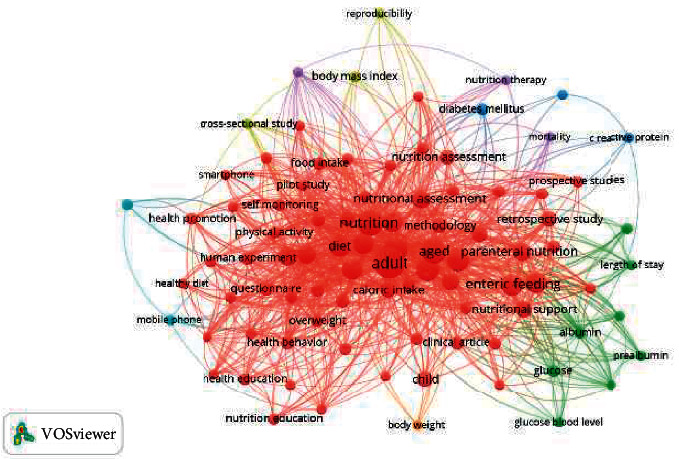
Network visualization of each cluster.

The author's keywords were diagrammed with VOSviewer, a software tool for constructing and visualizing bibliometric networks.  Figure 7 presents a network visualization and overlay visualization.

Figure 7 illustrates the network visualization of each cluster using a color code. In this case, the colors used are red, green, yellow, blue, orange, purple, and navy. The network visualization depicted in Figure 7 represents all publications with underlying themes that frequently appeared in this study. The VOSviewer software found a total of 2066 keywords, 99 of which met the data analysis criteria from 205 Scopus-indexed documents published between 2007 and 2021 if filtered by at least seven terms. VOSviewer was used for bibliometric mapping of dietary self-monitoring, divided into 7 clusters, as shown in Table 1.

Cluster 1 is expected to contain the documents related to topics of interest in attitude to health, behavior change, body mass, and caloric intake. Meanwhile, the albumin blood level, glucose blood level, and prealbumin are located in Cluster 2. Cluster 3 deals with C reactive protein, diabetes mellitus, and triacylglycerol, while Cluster 4 relates to body mass index and reproducibility. Cluster 5 is also associated with algorithms, mortality, and nutrition therapy. Cluster 6 contains mHealth and mobile phones. Last, Cluster 7 relates to body weight. Table 1 can be the key to the theme of each cluster.

The researchers chose two themes that are related to the present study: attitude to improved dietary behaviors and mobile health applications (Table 2). These two themes are the most similar to this research topic. A reported study by West et al. aims to identify the change of behavior related to the use of diet and nutrition application [23]. Moreover, Turner-McGrievy et al.'s study compares self-monitoring of physical activity and nutritional intake using traditional methods to that using mobile apps [32].

A study found that over the course of six months, participants who followed a behavioral weight loss program that encouraged unrestricted consumption of a variety of low-energy-dense foods lost 7.9% of their initial body weight and saw significant improvements in blood pressure, aerobic stamina, quality of life, happiness, sleep, perceptions of hunger, and cravings for high-calorie and high-fat foods [34]. Weight loss is consistent with clinically substantial improvements in health risk variables in other intensive, in-person behavioral therapies [35]. Given the significant link between self-monitoring and weight loss [36], outcome expectations that encourage self-monitoring and increase self-efficacy for weight-loss-promoting actions should result in more weight loss [37]. It can be said that the participants' behavior can change according to a healthy self-monitoring diet.

Individuals may find smartphone apps to be a unique and practical technique of nutritional self-monitoring, and they can be customized to carry out educational and other health-related behavior change interventions [19]. Moreover, participants who used a commercially available app recorded entire days of dietary data more regularly than those who used paper and pencil [19]. Maybe, this is because people carry smartphones for a variety of other reasons, thus using smartphone-based solutions may prove to be a more straightforward and scalable intervention technique compared to traditional tracking methods [38]. According to the findings of West et al. research [23], diet or nutrition apps use IDs linked to diet-related behavior modification. They reported that diet and nutrition applications that focus on improving motivation, desire, self-efficacy, attitudes, knowledge, and goal-setting are becoming increasingly popular.

### 3.5. Citation Documents

Table 3 categorizes the articles based on the number of citations received. This is applied to map the research of publications that are most relevant to the issue of dietary self-monitoring. The number of article citations obtained across the entire database is the international source. Table 3 shows the ten most globally and locally cited publications from 2007 until 2021. It is essential to mention that the rankings are based on local citations.

Table 3 describes the researchers' analysis of the global citations in the articles. The international source is the total number of article citations obtained across the entire Scopus database. The three dominant cited articles were “Comparison of Traditional versus Mobile App Self-Monitoring of Physical Activity and Dietary Intake among Overweight Adults Participating in an mHealth Weight Loss Program” (*n*: 247) in 2020 [25]; the article by Hebden et al. [26] entitled “Development of Smartphone Applications for Nutrition and Physical Activity Behavior Change” (*n*: 128) in 2022; and the article entitled “Dietary Self-Monitoring, But Not Dietary Quality, Improves with Use of Smartphone App Technology in an 8-Week Weight Loss Trial” by Wharton et al. [19] with 110 citations in 2022.

### 3.6. Trending Topics

Table 4 presents the trending topics. The size or diameter represents the frequency of phrases provided by the author with keywords based on the bibliography periodicity parameters of at least five words each year. In 2007–2021, terms such as nutrition (*n* = 2294), health (*n* = 1734), food (*n* = 898), study (*n* = 891), and patients (*n* = 817) can be observed in Table 4. Furthermore, recent studies have shown that, in the face of this research, researchers' experience found self-monitoring dietary and physical activity [29, 34, 45–47], healthy diet and lifestyle [45], and mobile application of dietary self-monitoring [46, 48–50].

Table 5 reveals the Pearson correlation among the total dietary scores of the assessment method, quality, data collection, analysis, feedback, records, and self-monitoring. The top results showed that the overall dietary scores were positively associated with assessment method, quality, and data collection, with correlation values ranging from 0.78 to 0.73, according to the point-biserial correlation coefficient. The limitation of this study is that the results are subjective according to the authors' knowledge and the topic they want to discuss.

## 4. Conclusion

In conclusion, many people have become aware of their health. Moreover, they are also informed how to live a healthy lifestyle. Many studies discuss dietary self-monitoring, and the research on this topic increased in 2017 and started to include mobile applications. Based on the analysis that was carried out using VOSviewer and NVivo, the results of clusters and trending topics from the study were obtained. The researchers chose two cluster themes related to this research: attitudes to improved dietary behavior and mobile health applications. Most of the research related to these themes aims to identify changes in healthy lifestyle behavior with mobile applications that are considered effective in dietary self-monitoring. Furthermore, recent studies have shown that, in the face of this research, researchers experience found self-monitoring dietary and physical activity, healthy diet and lifestyle, and mobile application of dietary self-monitoring. Based on the results of this research, the authors recommend, for future research, the development of a nutrition mobile application that helps people self-monitor their diet based on their lifestyle behavior.

## Figures and Tables

**Table 1:** The clusters of keyword analysis.

Cluster	Items	Total	Percentage (%)
**Cluster 1**	Adolescent, adult, aged, attitude to health, behavior change, body mass, caloric intake, child, clinical article, clinical trial, comparative study, controlled clinical trial, diet, diet records, diet therapy, dietary intake, energy intake, enteral nutrition, enteric feeding, exercise, feeding behavior, food intake, food preference, health behavior, health education, health knowledge, health promotion, healthy diet, human experiment, intensive care unit, major clinical study, malnutrition, methodology, middle-aged, mobile application, mobile applications, nutrition, nutrition assessment, nutrition science, nutritional status, nutritional support, obesity, outcome assessment, overweight, parenteral nutrition, physical activity, pilot study, practice guideline, priority journal, procedures, prospective study, protein intake, questionnaire, randomized controlled, retrospective study, risk factor, self-care, self-monitoring, smartphone, survey and questionnaire, treatment outcome, weight loss, weight reduction, young adult	71	78

**Cluster 2**	Albumin, albumin blood level, glucose, glucose blood level, hospitalization, length of stay, postoperative care, prealbumin	8	8.8

**Cluster 3**	C reactive protein, diabetes mellitus, triacylglycerol	3	3.3

**Cluster 4**	Body mass index, cross-sectional study, reproducibility	3	3.3

**Cluster 5**	Algorithm, mortality, nutrition therapy	3	3.3

**Cluster 6**	mHealth, mobile phone	2	2.2

**Cluster 7**	Body weight	1	1.1

**Table 2:** Themes of clusters in keyword analysis.

Cluster themes	Author	Purpose	Finding
Attitude to improved dietary behaviors	Joshua H West et al., 2017	The purpose of this study was to identify which behavior change mechanisms are associated with the use of diet and nutrition-related health apps and whether the use of diet- and nutrition-related apps is associated with health behavior change.	Study findings indicate that diet/nutrition apps are associated with diet-related behavior change. Hence, diet- and nutrition-related apps that focus on improving motivation, desire, self-efficacy, attitudes, knowledge, and goal-setting may be useful [23].

Parameters for disease diagnosis	Den Braber et al., 2019	An ideal application would track food intake, physical activity, glucose levels, and medication use and then combine the data to give patients and healthcare providers insight into these elements and the impact of lifestyle on glucose levels in everyday life.	This research focuses on the needs for the initial iteration of the diameter, which are focused on gathering data and providing insight to patients. It is critical to collect lifestyle and glucose data rapidly to construct future versions of the diameter, including a personalized data-driven coaching module [28].

Noncommunicable diseases (NCD)	Richardson et al., 2021	This study aims to see if an abridged dietary self-monitoring method in T2D patients, in which only carbohydrate-containing foods are recorded in a diet tracker, is feasible.	A simplified dietary self-monitoring strategy may not be possible, especially for people unfamiliar with carbohydrate-containing meals. Despite these findings, this study contributes to the little literature that examines alternatives to more intense dietary self-monitoring for T2D management [29].

Methods	Prudhon et al., 2011	It provides an algorithm for analyzing nutritional and mortality survey reports using systematic and comparable criteria to identify a wide range of errors that could lead to sample, response, or measurement biases and rate the overall quality of the survey.	The intra-class correlation coefficient for mortality surveys was 0.79, while for nutrition surveys, it was 0.78. For mortality and nutrition surveys, the total median quality score and range of around 100 surveys completed in Darfur were 0.60 (0.12–0.93) and 0.675 (0.23–0.86), respectively. They vary depending on the surveying organization, with no discernible trend over time [30].

Nutrition algorithms	Sun et al., 2012	It contributes to understanding the human factors that determine diets, eating patterns, and lifestyle choices by describing specific task force actions and findings in Asian countries. It also discusses the impact of transcultural factors on the adaptability of current evidence-based CPGs for diabetes-specific nutrition therapy and their implementation in Asian nations.	An international task team created a transcultural diabetes-specific nutrition algorithm that breaks down complex diabetes rules into a simple, customizable structure. To address the demands and preferences of afflicted patients, the Asian adaption integrates regional variances in lifestyles, diets, and customs [31].

Mobile health applications	Turner-McGrievy et al., 2017	The purpose of this study was to compare traditional and mobile app self-monitoring of physical activity and dietary intake.	The study findings point to the potential benefits of mobile monitoring methods during behavioral weight loss trials. Future studies should examine ways to predict which self-monitoring method works best for an individual to increase adherence [32].

Body mass index	Chen and Tseng, 2010	It aims to determine the marginal effects of food intakes, health behaviors, and nutrition knowledge on the overall BMI distribution across individuals.	Evidence suggests that calories, oleic acid, and cholesterol raise BMI, but fiber, calcium, and sodium have the opposite impact. Protein decreases BMI in females who are overweight or obese. Vitamin C lowers BMI in underweight and mildly to severely obese males. Jogging reduces BMI. However, drinking enhances BMI in nonobese people. Nutrition knowledge lowers BMI in males whose BMI is in the optimal weight to slightly overweight ranges, whereas this effect is minor in females [33].

**Table 3:** Top article citations.

Title	Author and year	Source	Cited by
Comparison of Traditional versus Mobile App Self-Monitoring of Physical Activity and Dietary Intake among Overweight Adults Participating in an mHealth Weight Loss Program [25]	Turner-McGrievy et al., 2013	Journal of the American Medical Informatics Association	247
Development of Smartphone Applications for Nutrition and Physical Activity Behavior Change [26]	Hebden et al., 2012	JMIR Research Protocols	128
Dietary Self-Monitoring, But Not Dietary Quality, Improves with Use of Smartphone App Technology in an 8-Week Weight Loss Trial [19]	Wharton et al., 2014	Journal of Nutrition Education and Behavior	110
Popular Nutrition-Related Mobile Apps: A Feature Assessment [39]	Franco et al., 2016	JMIR mHealth and uHealth	88
Factors Related to Sustained Use of a Free Mobile App for Dietary Self-Monitoring with Photography and Peer Feedback: Retrospective Cohort Study [40]	Helander et al., 2014	Journal of Medical Internet Research	79
There Are Thousands of Apps for That: Navigating Mobile Technology for Nutrition Education and Behavior [41]	Hingle and Patrick, 2016	Journal of Nutrition Education and Behavior	68
Demographic and Socioeconomic Disparity in Nutrition: Application of a Novel Correlated Component Regression Approach [42]	Alkerwi et al., 2015	BMJ Open	56
Controlling your “App”etite: How Diet and Nutrition-Related Mobile Apps Lead to Behavior Change [23]	West et al., 2017	JMIR mHealth and uHealth	52
Multivariate Techniques and Their Application in Nutrition: A Metabolomics Case Study [43]	Kemsley et al., 2007	British Journal of Nutrition	48
Application of the Nutrition Functional Diversity Indicator to Assess Food System Contributions to Dietary Diversity and Sustainable Diets of Malawian Households [44]	Luckett et al., 2015	Public Health Nutrition	39

**Table 4:** Trending topics of keywords.

Word	Length	Count	Weighted percentage (%)	Word	Length	Count	Weighted percentage (%)
Nutrition	9	2294	0.98	Care	4	532	0.23
Health	6	1734	0.74	Self	4	525	0.22
Food	4	898	0.38	Analysis	8	515	0.22
Study	5	891	0.38	Mobile	6	509	0.22
Patients	8	817	0.35	Obesity	7	509	0.22
Dietary	7	749	0.32	Diabetes	8	508	0.22
Diet	4	736	0.31	Data	4	492	0.21
Weight	6	734	0.31	Clinical	8	489	0.21
Nutritional	11	649	0.28	Disease	7	462	0.20
Nutrition	9	2294	0.98	Intake	6	453	0.19

**Table 5:** Relation of the dietary keyword.

	Code A	Code B	Pearson correlation coefficient
Dietary self-monitoring	Dietary	Assessment methods	0.796755
	Dietary	Quality	0.776247
	Dietary	Data collection	0.732334
	Dietary	Analysis	0.64684
	Dietary	Feedback	0.617656
	Dietary	Records	0.572259
	Dietary	Self-monitoring	0.505584

## Data Availability

The data used in this research were adopted from Scopus database.
